# Deictic and Propositional Meaning—New Perspectives on Language in Schizophrenia

**DOI:** 10.3389/fpsyt.2017.00017

**Published:** 2017-02-10

**Authors:** Vitor C. Zimmerer, Stuart Watson, Douglas Turkington, I. Nicol Ferrier, Wolfram Hinzen

**Affiliations:** ^1^Department of Language and Cognition, University College London, London, UK; ^2^Institute of Neuroscience, Newcastle University, Newcastle upon Tyne, UK; ^3^ICREA (Institució Catalana de Recerca i Estudis Avançats), Departament de Traducció i Ciències del Llenguatge, Universitat Pompeu Fabra, Barcelona, Spain; ^4^Department of Philosophy, Durham University, Durham, UK

**Keywords:** schizophrenia, positive symptoms, language, grammar, cognitive behavioral therapy

## Abstract

Emerging linguistic evidence points at disordered language behavior as a defining characteristic of schizophrenia. In this article, we review this literature and demonstrate how a framework focusing on two core functions of language—reference and propositional meaning—can conceptualize schizophrenic symptoms, identify important variables for risk assessment, diagnosis, and treatment, and inform cognitive behavioral therapy and other remedial approaches. We introduce the linguistic phenomena of deictic anchoring and propositional complexity, explain how they relate to schizophrenic symptoms, and show how they can be tracked in language behavior.

## Language and Thought in Schizophrenia

Human thought is best expressed through language output. No other type of behavior is able to capture its complexity with as little ambiguity or effort. It is therefore no surprise that primary symptoms of schizophrenia, in particular thought disorder and delusions, are inseparable from, and characterized by, linguistic behavior. Thought disorder is characterized and diagnosed by “disordered speech” (1), and it is through language that delusional thought is expressed. Schizophrenia appears to be associated with specific language profiles (2–7), particularly with semantic impairment in thought disorder and poverty and disruption of grammatical structure in schizophrenia at large. Moreover, the demonstration premorbidly of linguistic deviation may predict the transition to psychosis in high-risk individuals (8, 9). Language integrates a wide range of cognitive functions: perception, mind-reading, intention, memory, executive functioning, prediction, and motor control. This integration of cognitive systems has long been seen to be important in psychotic disorders. For instance, Bleuler believed that a “disorder of associations” between cognitive functions may underlie schizophrenia (10). Thus, failure of one or several of these cognitive processes, or their integration, may engender the symptoms of schizophrenia and may be revealed by language. The contention that schizophrenia presents with a disorder of language is supported by biological evidence: language draws from a large, connected network that extends well beyond Broca’s and Wernicke’s areas and includes right hemisphere and subcortical structures, including striatal areas which are relevant for motor speech control, semantic and grammatical processes (11), and which are affected in schizophrenia (12, 13). The white matter tracts which support language function are also involved in other aspects of cognition including memory and executive function (14) and are abnormal in schizophrenia (15). Genes that mediate linguistic behavior are associated with schizophrenia (16); a polymorphism in the FOXP2 gene for instance associates with schizophrenia (17), with poverty of speech, and possibly with auditory verbal hallucinations (18, 19).

In this article, we introduce one approach to use the tools of linguistics to help identify specific language variables which may advance clinical practice both in diagnosis and in intervention. Despite early associations with aphasia (20), there are substantial differences between associated symptoms (21, 22). In particular, processing verb argument structure, affixation, or retrieval of complex, rare, and/or abstract words appear comparatively intact in schizophrenia.

There has been much attention to single word processing in schizophrenia, especially with regards to semantic priming. Priming refers to the phenomenon that the response to a word (e.g., “cat”) is faster when preceded by a related word (e.g., the semantically related “dog,” or phonologically related “mat”). This priming effect has been repeatedly shown to be enhanced in schizophrenia, with evidence of increase in thought disorder (23, 24). One pharmacological study has shown that priming effects are subject to dopaminergic modulation (25). It has been argued that enhanced priming in schizophrenia is consequent on increased automatic spreading of activation in the lexical–semantic system (26). Language production too provides evidence for pathological activation patterns: in a verbal fluency task (“name as many animals as you can”), people with schizophrenia and their unaffected siblings produced more words than controls and the words were, semantically, more closely related (27). These studies have provided valuable insight into possible disruptions of neural connectivity, especially in the lexicon. Because words are considered very basic units in language, it may be tempting to assume that they lie at the basis of language processing. However, word selection occurs in tandem with selection of grammatical frames, and as a result, processing of words in such isolation is virtually absent from natural language use. Words appear in grammatical and communicational contexts. We will focus on grammar, and in particular two inherent aspects of language structure: deictic anchoring and propositional meaning.

In this article, we treat language primarily as an indicator of mental health issues. We acknowledge that cognitive processes in schizophrenia are atypical in non-verbal domains, as experiments on prepulse inhibition (28, 29) and visual processing (30), among other work, suggest. However, we take seriously the possibility that language contributes to shaping higher level thought. In our discussion of behavioral intervention toward the end of the article, we make assumptions that treating language use could at least influence the way thought is structured.

As a final note of introduction, people with a diagnosis of schizophrenia form a highly heterogeneous group even when grouped according to symptom labels. In this paper which aims to introduce new perspectives, it is impossible to do justice to the entire spectrum. It will take more empirical work to test the ideas described herein.

## Deictic Anchoring

Deictic anchoring is an inherent part of the process by which we make references to aspects in the world including entities, events, locations, and time. Consider a news report about a road traffic incident in which the reporter says: “A jeep crashed into a barrier.” The meaning is deictically anchored firstly by references to entities, e.g., “a jeep” or “a barrier,” and we know from the context that it is one particular jeep and one particular barrier (as opposed to the same phrase in the generic statement “a jeep is a type of car”). The event (“crashed”) is anchored in time as being in the past relative to the speech act. Healthy deictic anchoring further expands to having a sense of who the speaker is and who that speaker is addressing. As a listener, I know that the reporter is not talking about a car crash I had last year and that she is not talking exclusively to me.

Psychotic episodes have been reframed as a disruption of deictic anchoring (31). With regard to our example news report, someone with disturbed deictic anchoring may believe that the reporter is directly and specifically talking to him or her about a car accident that (s)he experienced last year. Such self-referential beliefs are characteristic of schizophrenia (1). Thoughts too are anchored to who is thinking them and to what they relate. Under that framework thought control and thought insertion (someone else thinks my thoughts), thought broadcast (my private thoughts are accessed by someone else), and auditory verbal hallucinations (in which my own language is perceived as the speech of someone else which is directed at me or about me) can be conceptualized as a disruption of deictic anchoring (31, 32).

Such a disruption would manifest in language behavior. Crucial deictic information is conveyed in nouns or noun phrases, e.g., “a man” and “that red car.” Pronouns also serve deictic anchoring. Fineberg et al. (33) found increased first-person (self-referential) pronoun use in psychosis. In another study, pronoun use also distinguished people with schizophrenia from people with mood disorder (34). Watson et al. found that, in people with high genetic loading for schizophrenia, participants who transition to schizophrenia can be predicted by their increased use of second-person pronouns (9). This linguistic profile was stable at two separate assessments 18 months apart and predated other diagnostic symptoms.

## Propositional Meaning

For linguistic structures to be complete, deictic anchoring must be used to form propositions. Propositions are statements about the world which can be true or false. They form the basis of human reasoning and determine our views, selfhood, and actions. In linguistic behavior, propositions emerge in complete sentences (or clauses). A noun such as “jeep,” by itself, is not propositional. It refers to a general concept, but it contains no statement. Which jeep? What happened, is happening, or will happen to it? Does it even exist? It is the full sentence, in a context, that forms a proposition. “A jeep crashed into a barrier” establishes a topic/subject (“a jeep”) and makes a conceptually verifiable statement about it.

Increasing propositional complexity correlates with increasing grammatical complexity. Consider the sentences “Sarah realizes that a jeep crashed into a barrier” and “Sarah thinks that a jeep crashed into a barrier.” Each of these sentences contains an embedded clause, e.g., “[Sarah realizes [that a jeep crashed into a barrier]]” (square brackets indicate clauses, one of which is here hierarchically embedded in another). The embedded content is dependent on the verb in the superordinate clause. Mental state verbs can be categorized as factive (e.g., “realize,” “regret,” and “know”) or non-factive (e.g., “believe,” “think,” and “assume”). For a factive sentence such as “Sarah *realizes* that a jeep crashed into a barrier” to be true, the jeep must have crashed into a barrier and Sarah must be certain about it. For the non-factive sentence, “Sarah *thinks* that a jeep crashed into the barrier” to be true, it does not actually matter whether the accident happened. Only Sarah’s representation of the world is important. Other types of embedding infer causality (“because”) or temporal relationships (“after”). Clausal embedding, and to a smaller degree the juxtaposition of clauses (e.g., connecting clauses with “and” or “or”), introduce complex relationships between propositions. Kuperberg (7) notes that in people with schizophrenia, cortical activity to semantic abnormalities in sentences is particularly small compared to controls if interpretation requires integration of several sentences.

Delusions and thought disorder can be considered disruptions of propositional meaning. The affected individual considers as true propositions that others would reject with certainty, or, if thought disordered, may altogether lose the ability to form coherent propositions. A reduced capacity to entertain complex propositions in schizophrenia may underlie formal thought disorder and the generation of delusions (32).

Theory of Mind (ToM) tasks, including those in studies showing ToM impairment in schizophrenia, commonly make use of non-factive embedding [e.g., “When John comes back for his cigarettes, how many does he think he has left?” (35)]. In child development, a relationship between emergence of clause embedding and ToM ability has been proposed (36). Similarly, impaired ToM in schizophrenia (37) may be related to decreased language complexity. Propositional complexity may also distinguish prodromal stage overvalued ideas (38) from delusions. The former turn out to be more complex [e.g., the non-factive embedding in “[People look confusing] … [they’re almost like [they’re made up]]” (39)], while delusional statements can be simpler [e.g., “[I have a million dollars]” (40)]. Klaus Conrad described the onset of a delusion as the loss of ability to transcend an experience and see it with the eyes of others (41). At a linguistic level, this cognitive restructuring can be described as a loss of propositional complexity.

In thought disorder, the ability to express coherent propositions can be severely impaired. Sentences are structurally incomplete, abandoned by the speaker, or contain grammatical errors which severely hinder their interpretation. “Anna” is a 58-year-old lady with a 30-year history of treatment resistant schizophrenia characterized by severe thought disorder, affective incongruity, low motivation, poor self care, and delusions of grandeur. In the following excerpt, the therapist probes after she says that she worked as a judge:

**Table:** 

Therapist:	You were working as a judge?
Anna:	Yes.
Therapist:	Whereabouts did you work as a judge?
Anna:	England.
Therapist:	In England.
Anna:	Judge Supreme, agnostic, when I left, and move into the royal. Biggest judge in the world.

The patient’s final statement does not contain a single complete clause. “Judge Supreme” and “agnostic” are a noun and an adjective, respectively, produced in grammatical isolation. “When I left” is a dependent clause and as such cannot be interpreted if not connected to another clause (e.g., “[She was still there [when I left]]”). “Move into the royal” is not a clause since it is missing its subject. We can speculate that this subject could be the preceding pronoun “I,” but in this case, the verb would lack a past tense marker (“moved”). “Biggest judge in the world” is a complex, but isolated, noun phrase. Propositions are difficult to extract under such circumstances. Note that Anna is a severe case and that the majority of individuals with thought disorder will not present disruptions of such degree.

## The Linguistics of Behavioral Intervention

We have discussed how language behavior can contribute to identifying the risk of developing schizophrenia and how it is essential to detecting schizophrenic symptoms. In this section, we wish to discuss language as a mediator of cognitive change in schizophrenia, especially in talk-based therapies such as cognitive behavioral therapy. Language is the medium through which the therapist puts a patient’s thought process and assertions into focus with a view to being reframed (42). Moreover, we consider here whether cognitive restructuring can occur as a consequence of the therapist helping the patient to change their language behavior, for instance, by first establishing coherent deictic anchoring and propositional meaning. This is crucial, since without deictic anchoring, no meaningful interaction is possible, and without propositional meaning, there is literally nothing to discuss. Going back to Anna, we can see the therapist trying to form propositions on the basis of her structurally impoverished output. The therapist is inquiring about a process she calls “sight and mind painting”:

**Table:** 

Therapist:	What does the sight and mind painting involve?
Anna:	Ah, you can do it on the camera, on the chair and the top of a … a door, say, yes, flown in by radar.
Therapist:	So you put together a composition …
Anna:	Yes.
Therapist:	And it’s passed by radar.
Anna:	Yes.
Therapist:	And … how does the radar work? Is there any kind of equipment?
Anna:	Yeah, yeah, I’m not sure about that.
Therapist:	So there might be equipment? Right?
Anna:	Yeah.
Therapist:	So how, how do you process it, you’ve got that composition there …
Anna:	There are, there have canvasses, down in London and somehow, I see what I’m looking at, that becomes a painting. The painting is then transferred.
Therapist:	The canvasses down in London, somehow your composition is transferred there?

Treatment of delusions can involve turning the delusional assertion (“I am John the Baptist”) into a non-delusional one (“I feel I am John the Baptist”). The introduction of complexity loosens the force of the cognitive distortion and represents the delusion not simply as fact but as a content for possible denial and scrutiny. There is corresponding work in depression: Zinken et al. (43) found that the complexity of clausal connectivity and embedding was a significant predictor for how well a depressed individual would respond to therapy. The authors assumed that richer grammatical systems represented the individual’s ability to not just maintain simple negative propositions (“[I feel bad] [and I can’t sleep]”) but to put them into a context that would allow further insight (“[I feel bad [because I can’t sleep]]”) and thereby cope or even gain control over their negative thought processes. Syntactic priming, the tendency to reproduce specific language structures that one is exposed to (44, 45), suggests that the therapist’s language use may be critical. A three-step linguistic therapy model therefore emerges to (i) establish deictic anchoring, (ii) establish propositional meaning, and (iii) increase propositional complexity. This notion requires the traditional belief that language is simply a way of expressing thought to be challenged; it relies instead on a more nuanced understanding of a closer interdependence of thought and language (46). It also requires rigorous testing. It needs to be determined whether it is possible for therapists to increase the complexity of the language used to report the delusional beliefs, and whether such a linguistic change will reduce the resistance of the delusion to disconfirmatory bias. Finally, the language variables relevant to predicting and identifying schizophrenia may serve as outcome variables which track cognitive change as the result of behavioral or pharmacological interventions.

## Toward a Cognitive Profile of Schizophrenic Symptoms

Language has become a new frontier in clinical research, not only in schizophrenia but also in other pathologies such as dementias (47, 48). Language variables can be obtained non-invasively and without requirement for expensive technologies. Their analysis can be automated (8, 49, 50). They are quantitative and continuous, and may reveal subtle cognitive signs long before recognized onset. It appears that the variables that most critically inform diagnosis and support therapy vary from pathology to pathology. In the case of schizophrenia, noun phrase use and degrees and types of clausal combination and embedding appear particularly relevant. Linguistic research should be used to complement research on other cognitive processes such as memory, attention, inhibition, and visual perception.

## Author Contributions

VZ wrote the manuscript and implemented suggestions from the co-authors. SW co-wrote the manuscript and contributed to its conception. DT provided language samples, contributed to the conception of the manuscript, and helped drafting the work. INF contributed to the conception of the manuscript and helped drafting the work. WH is the project PI and originator of the basic concepts in the manuscript. He helped drafting the work.

## Conflict of Interest Statement

The authors declare that the research was conducted in the absence of any commercial or financial relationships that could be construed as a potential conflict of interest. The reviewer AP and handling Editor declared their shared affiliation, and the handling Editor states that the process nevertheless met the standards of a fair and objective review.
